# OA14.05. Hypnosis for hot flashes: results from a randomized clinical trial and future directions

**DOI:** 10.1186/1472-6882-12-S1-O57

**Published:** 2012-06-12

**Authors:** G Elkins, W Fisher, A Johnson

**Affiliations:** 1 Baylor University, Waco, USA

## Purpose

Hot flashes are a significant clinical problem for many women. Currently there are limited options to hormone replacement therapy as non-hormonal pharmacological agents are associated with only modest activity and many adverse side effects. Hypnosis is one mind-body therapy that seems particularly promising for treating hot flashes and was investigated in the present study. This study examined the efficacy of hypnosis in reducing both self-reported and physiologically determined hot flash frequency and severity among post-menopausal women.

## Methods

One-hundred and seventy post-menopausal women with moderate to severe hot flashes were randomly assigned to either a 5-session hypnosis intervention or a 5-session structured-attention control condition. All sessions were provided consistent with a treatment manual and all therapists were trained to criteria for consistency and treatment fidelity. Primary outcome measures were self-reported hot flash frequency and severity (determined via daily diaries) and physiologically monitored hot flashes (determined via sternal skin conductance). Physiological assessment of hot flashes were made using 24-hour recordings of sternal skin conductance. Measures were obtained at baseline, at the end of the five weeks intervention, and at 12 week follow-up.

## Results

Results demonstrated that hot flash scores (self-report of frequency and severity of hot flashes) for the participants that received the therapist delivered hypnosis intervention decreased by approximately 70% at 5 weeks and continued to decline to approximately 80% at the 12 week follow-up. Physiologically assessed hot flashes demonstrated a 50% reduction at 5 weeks and approximately 60% reduction at 12 weeks for participants in the therapist delivered hypnosis condition.

## Conclusion

To our knowledge, this is the first study to demonstrate a clinically significant reduction in physiologically measured hot flashes using a hypnosis intervention. This study has important implications for women experiencing hot flashes who are contraindicated for hormone replacement therapy.

